# Please Do Something: What Caregivers for Family Members with FTD Need

**DOI:** 10.1093/geroni/igab046.3517

**Published:** 2021-12-17

**Authors:** Samantha Smith, Leslie Tran, Allison Lindauer

**Affiliations:** 1 Oregon Health & Science University, Portland, Oregon, United States; 2 Oregon Health Sciences University, Portland, Oregon, United States

## Abstract

Frontotemporal dementia (FTD) often presents with pronounced behavioral symptoms that contribute to family Care Partner (CP) burden and psychological strain. FTD-specific interventions that support the unique challenges of FTD-CPs are lacking. The present focus group study (Phase 1), elicited feedback from twelve CPs of persons with FTD on the multi-component video-based STELLA intervention (Support via TEchnology: Living and Learning with Advancing ADRDs), to inform the revision and adaptation of STELLA for FTD-CPs (Phase 2). Using Thomas’s (2006) analytic approach to evaluation data, the investigators reviewed the raw text from two focus groups and used an inductive approach to create categories that informed future STELLA adaptation and revision. To address trustworthiness, each investigator independently analyzed the transcripts and CP-annotated STELLA booklets. Six commonalities emerged. Three address the caregiver experience: Burden and living with complex behaviors; Difficulties in getting a diagnosis; and Barriers to participation. The other three reflect specific intervention adaptations: Make a Roadmap, STELLA-FTD Structure, and Booklet edits. The focus group findings offer practical suggestions to create a revised STELLA intervention to address the needs of families living with FTD. The suggestions are nested in the intense caregiving experience of living with complex behavioral symptoms, feeling burdened, isolated, and “living in darkness.” Both the investigators and caregivers appreciate the difficulty in crafting an intervention that meets the needs of all families living with FTD, but the caregivers encouraged the team to develop “something”: “…You won't be able to solve every problem or meet every need… [but] please, please do something.”

